# Paired RNA profiling of circulating small extracellular vesicles links survival in ALS to reactive glial - vascular programs

**DOI:** 10.64898/2026.09.08.749915

**Published:** 2026-09-14

**Authors:** Jonathan S. Weerakkody, Finja Bokstaller, Uma Sthanu, David Pitt

## Abstract

Circulating RNA can capture molecular variation associated with amyotrophic lateral sclerosis (ALS) progression, but biofluid heterogeneity makes biologically organized signals difficult to recover. Here, we paired microRNA (miRNA) and messenger RNA (mRNA) profiles from glutamate–aspartate transporter (GLAST)-positive small extracellular-vesicles (sEVs) to estimate survival time and identify survival-associated programs. In 45 participants with ALS and 15 controls, biologically constrained multi-omic factor analysis identified a program in which reciprocal miRNA–mRNA states among target-supported pairs tracked survival. The miRNA arm was then evaluated in an independent total-plasma cohort of 248 participants with ALS, refining a five-miRNA panel that added prognostic information beyond functional decline and neurofilament light chain, particularly over longer survival horizons. The mRNA arm was evaluated across 586 cortical profiles from 308 donors and 527,261 nuclei from 69 donors, identifying a five-gene core associated with glial reactivity, vascular programs and reduced myelinating identity. Together, these findings establish a framework for integrating regulatory RNA layers in circulation to identify clinically relevant programs and relate them to disease-relevant cellular states.

## Full Text Availability

The license terms selected by the author(s) for this preprint version do not permit archiving in PMC. The full text is available from the preprint server.

